# Genetic polymorphisms between altruism and selfishness close to the Hamilton threshold *rb* = *c*

**DOI:** 10.1098/rsos.160649

**Published:** 2017-02-08

**Authors:** Richard M. Sibly, Robert N. Curnow

**Affiliations:** 1 School of Biological Sciences, University of Reading, Whiteknights, Reading RG6 6AS, UK; 2 Department of Mathematics and Statistics, University of Reading, Whiteknights, Reading RG6 6AX, UK

**Keywords:** Hamilton's rule, population genetics, stable polymorphisms

## Abstract

Genes that in certain conditions make their carriers altruistic are being identified, and altruism and selfishness have shown to be heritable in man. This raises the possibility that genetic polymorphisms for altruism/selfishness exist in man and other animals. Here we characterize some of the conditions in which genetic polymorphisms may occur. We show for dominant or recessive alleles how the positions of stable equilibria depend on the benefit to the recipient, *b*, and the cost to the altruist, *c*, for diploid altruists helping half or full sibs, and haplodiploid altruists helping sisters. Stable polymorphisms always occur close to the Hamilton threshold *rb* = *c*. The position of the stable equilibrium moves away 0 or 1 with both increases in *c*, the cost paid by the altruist, and increasing divergence from the Hamilton threshold, and alleles for selfishness can reach frequencies around 50%. We evaluate quantitative estimates of *b*, *c* and *r* from field studies in the light of these predictions, but the values do not fall in the regions where genetic polymorphisms are expected. Nevertheless, it will be interesting to see as genes for altruism are discovered whether they are accompanied by alternate alleles for selfishness.

## 1. Introduction

Genes that in certain conditions make their carriers altruistic have recently been identified [1,2]. At the same time, in man, some 30–50% of the variation in willingness to help others is heritable [3–10] (but cf*.* [11]). This raises the possibility that genetic polymorphisms for altruism/selfishness exist in man and other animals.

The theoretical study of genetic polymorphisms between altruism and selfishness relies on single locus two-allele models that lead to recurrence equations for the frequencies of genotypes in successive generations [12–17]. Uyenoyama & Feldman [17] showed that equilibria are possible for a variety of mating systems, [14] showed that stable polymorphisms may occur in the case of full sibs, and [15] showed that stable polymorphisms may occur if benefits are non-additive or there is genetically based discrimination against selfish individuals [16]. These polymorphisms occur for values of *b*, *c* and *r* close to Hamilton's 1964 [18] threshold for the evolution of altruism, which is *rb* = *c*, where *r* = ½, ¼ or ¾ for full and half sibs and haplodiploid sisters, respectively. A wide variety of genetic models exists for studying altruism for other purposes (e.g. [14,19–24]) but these are not useful here because they assume weak selection, and this precludes the evolution of polymorphisms.

Here, we characterize the quantitative conditions that can give rise to stable genetic polymorphisms, by analysing the outcome of selection on genes that promote their carriers helping relatives. We do this for diploid altruists helping half or full sibs, and haplodiploid altruists helping sisters. Costs and benefits of help are measured as number of offspring lost or gained, but are here normalized so that individuals sacrificing all their personal reproduction pay a cost of one. Our analysis makes clear when and why stable genetic polymorphisms occur and shows how the positions of the stable equilibria depend on the cost and the cost/benefit ratio. We evaluate the available quantitative field estimates of *b*, *c* and *r*, collated and reviewed by Bourke [25], in the light of these predictions, and provide a brief discussion of the evidence that genetically based mixtures of selfishness and altruism occur in nature.

## 2. Models and theory

In our treatment here, we assume that helping/selfishness is controlled by two alleles, A and S, at a single locus in a diploid population. If A is dominant then AA and AS individuals are altruistic and help all their relatives with a specified coefficient of relatedness, whereas SS individuals are selfish and help no one. Conversely, if A is recessive then only AA individuals are altruistic. Our models assume that selection happens on juveniles before mating, and adults mate at random in a large population with no mutation, so that the dynamics is deterministic. Our method for deriving the recurrence equations that link the frequencies of genotypes in successive discrete generations is essentially that of [12–17]. In each generation, each individual that survives to breed produces a number of gametes and then dies. The number of gametes produced gives its fitness, after normalization so that in the absence of altruism fitness is 1. In studying altruism, it is helpful to conceptually segregate the population into sets of individuals sharing the same genotype and fitness, because the fitness of an individual depends not only on its own genotype but also on the genotypes of others who may help it, thus boosting its fitness. Let *g_j_* be the relative frequency of individuals of the *j*th set before selection, and let their fitness, relative to the average population fitness, be *v_j_*. The relative frequency of the set *j* individuals after selection will be *g_j_v_j_*. This is the basis of the recurrence relationships we now derive.

The first step in the analysis is to compare the frequencies of the three genotypes in parents with the frequencies in their offspring after they have experienced selection and so are due to become parents of the next generation. Table 1 shows the benefits and costs from interactions between helpers and recipients that determine the incremental fitness of each individual. In the calculations below costs and benefits are assumed additive if individuals interact with more than one sib (see [15] for relaxation of this assumption) and the size of the sibship is then absorbed into the definitions of the benefit and cost constants, so that *b* and *c* represent the cumulative effects of the benefits and costs of all interactions with relevant relatives. The relative frequencies of the three genotypes AA, AS and SS in the parental generation will be written *U*, *V* and *W*, respectively, and the frequencies of the alleles A and S are *p* = *U* + ½*V* and *q* = *W* + ½*V*, respectively.

**Table 1.** Individual fitness payoffs from interactions between pairs of individuals sampled at random from an infinite population. The payoffs are specified relative to mutual selfishness. The strategies of focal individuals are specified in the first column, and the strategies of those they interact with in the second and third columns. Thus, the bottom row shows that an altruistic individual interacting with a selfish individual loses *c* fitness units, but receives a payoff of *b* − *c* when interacting with another altruist.

	interactant	
individual	selfish	altruistic
selfish	0	*b*
altruistic	−*c*	*b* – *c*

The recurrence equations linking the three genotypic frequencies in successive generations can now be calculated using the methods in the electronic supplementary material. The frequency of the AA genotype after selection, *U*′, is *g*_1_*v*_1_ + *g*_3_*v*_3_, and this is calculated from electronic supplementary material, table A1, for half sibs with A dominant as

2.1*a* $$U^{\prime }=\frac{Up(1+b-c)+\frac{1}{2}Vp(1+\frac{1}{2}b(1+p)-c)}{1+(b-c)(1-q^{2})}.$$

Similarly, *V*′ = *g*_2_*v*_2_ + *g*_4_*v*_4_ + *g*_6_*v*_6_ and *W*′ = *g*_5_*v*_5_ + *g*_7_*v*_7_ giving

2.1*b* $$V^{\prime }=\frac{Uq(1+b-c)+\frac{1}{2}V(1+\frac{1}{2}(1+p)b-c)+Wp(1+pb-c)}{1+(b-c)(1-q^{2})}$$

and

2.1*c* $$W^{\prime }=\frac{\frac{1}{2}Vq(1+\frac{1}{2}(1+p)b)+Wq(1+pb)}{1+(b-c)(1-q^{2})}.$$

From equations (2.1), the per generation change in the frequency of the A allele, Δ*p* = *U*′ + *V*′/2 −*U*− V/2, is

2.2 $$\Delta p=\frac{\left(1\text{/}4)pq^{2}(b-4c)+(1/8)bq(2pq-V\right)}{1+(b-c)(1-q^{2})}\;.$$

Note that as *p* = *U* + *V*/2 and *q* = 1 − *p*, equilibrium values of *p* or *q* can be found in terms of *b* and *c* by setting *U*′ = *U*, *V*′ = *V* and *W*′ = *W* in equations (2.1). Although it might be thought that determination of local stability would require a two-dimensional analysis in terms of changes in *U* and *W* (remember *V* = 1 − *U* − *W*), in fact a one-dimensional analysis is sufficient. To see this, note that the frequency of the AA genotype after random mating but before selection is $p_{t}^{2}$ in generation *t*, and so the change in a generation is $p_{t+1}^{2}-p_{t}^{2}=(p_{t+1}-p_{t})(p_{t+1}+p_{t})$. Therefore, the change in the frequency of AA near equilibria always has the same sign as the change in allele frequency, (*p_t_*_+1_ − *p_t_*). Similarly, the change in the frequency of SS at equilibria has the same sign as *q_t_*_+1_ − *q_t_* = *p_t_* − *p_t_*_+1_. Hence, the frequencies of the AA and SS genotypes (and by implication that of AS) return to equilibrium if and only if *p* does. Hence, the local stability or otherwise of the genotypic frequencies is as for the allele frequencies and so the local stability can be described in terms of one not two dimensions. Only if the perturbations to the equilibrium are large enough to take the allele frequencies beyond the unstable equilibrium and hence to fixation will the one-dimensional argument not hold.

The first term in the numerator of (2.2) leads to Hamilton's rule that Δ*p* > 0 if *b* > 4*c* but this ignores the contribution of the second term that is zero only if the genotypes of the parents are, despite selection, in Hardy–Weinberg equilibrium, *V* = 2*pq*. The coefficient of relatedness, *r*, fully describes the genotypes in the relative of an individual only if selection is so weak that the genotypes of any common ancestors are in Hardy–Weinberg equilibrium. Without this assumption a correct study of the evolution of altruism requires a specified genetic model, as in this paper and [12,14–17], calculating genotypic and not just allele frequencies.

Equations (2.1) and (2.2) hold whatever the strength of selection. Corresponding equations for S dominant are given in the electronic supplementary material, together with those for full sibs, which are special cases of equations given by [17] in their Table V, Model IIA and are also derived in [14]. The consequences of these equations will now be discussed.

## 3. Visualizing the effects of selection on alleles with specified effects

**Figure 1.**
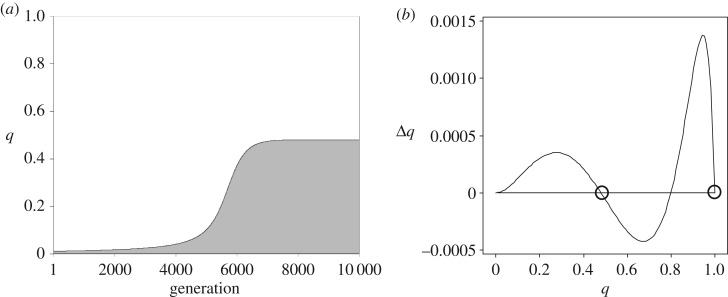
The spread of a recessive S allele in successive generations for half sibs for *b* = 3.7 and *c* = 1. Panel (*a*) shows the frequency of the S allele, *q*. Panel (*b*) shows the change in *q* per generation, Δ*q* = − Δ*p*, for all possible values of *q*. The positions of the stable equilibria are circled. The curves in (*a*) and (*b*) are drawn through discrete points obtained by iterative use of equations (2.1) and (2.2) updating genotype frequencies each generation from an initial Hardy–Weinberg equilibrium with *q* = 0.01. Thus, the curves depend on initial frequencies and the system is not, despite appearances, one-dimensional.

To help visualize the process of selection, we show an example in figure 1*a* of S alleles spreading into a population containing only A alleles. The calculations underlying these plots involve iterative use of equations (2.1). Although initial genotype frequencies affect the trajectories in figure 1*a* and electronic supplementary material, figure A1, trajectories with specified values of *b* and *c* were found to converge within a few generations whatever the initial genotype frequencies. So figure 1 and electronic supplementary material, figure A1, give a fair representation of the process of selection whatever the initial genotype frequencies.

The values of *b* and *c* in figure 1*a* were chosen such that *b* < 4*c*, so that according to Hamilton's rule for half sibs the S allele should spread to fixation. Figure 1*a* shows however that although the S allele does spread initially, it gets stuck at *q* = 0.48. Figure 1*b* shows that the rate of spread of the S allele per generation, Δ*q*, increases with *q* up to a maximum at *q* = 0.27 and then declines to zero at *q* = 0.48 (circled in figure 1*b*). So, for *q* < 0.48, the S allele increases in frequency, whereas for values of *q* between 0.48 and 0.80 the value of Δ*q* is negative, indicating that in this region the S allele declines. Thus, *q* = 0.48 represents an equilibrium stable to perturbations in the allele frequency. By contrast, *q* = 0.80 is an unstable equilibrium, and for *q* > 0.80 the S allele spreads to fixation. So figure 1*b* shows that although the initial spread of the recessive S allele follows Hamilton's rule, it does not spread to fixation and instead gets stuck in a stable equilibrium at an intermediate value of *q*.

Figure 1*b* can also be used to analyse the spread of dominant A alleles into homozygous S populations because when S is recessive, A is dominant. When A is rare, *p* is small and *q* = 1 − *p* is close to 1. Inspection of figure 1*b* shows that when *q* is close to 1, Δ*q* is positive. It follows that Δ*p*= −Δ*q* is negative. So the frequency of the A allele declines when *p* is close to 0, so a dominant A allele cannot enter a homozygous S population. This is what we expect from Hamilton's rule because the values of *b* and *c* were chosen such that *b* < 4*c*. However, if the frequency of the A allele were ever to get above *p* = 0.20 (so *q* < 0.80), the A allele would be selected towards the stable equilibrium at *p* = 0.52 (*q* = 0.48). Figure 1*b* can therefore be used to predict both the fate of recessive S alleles entering homozygous A populations and the fate of dominant A alleles entering homozygous S populations. So diagrams like that in figure 1*b* provide a key to understanding the spread of alleles.

Diagrams like figure 1*b* are shown for half and full sibs in electronic supplementary material, figure A1, for values of *b* and *c* chosen to show the range of values that can lead to polymorphisms. It appears from the graphs in electronic supplementary material, figure A1, that the conditions for alleles to spread initially are given exactly by Hamilton's rule. Thus, S alleles spread initially, Δ*q* > 0, in homozygous A populations if and only if *b* < *c*/*r*, and A alleles spread initially, Δ*p* > 0, in homozygous S populations if and only if *b* > *c*/*r*. However, for recessive alleles Hamilton's rule breaks down close to the threshold *rb* = *c*. Although the initial spread of recessive alleles follows Hamilton's rule, they do not spread to fixation and instead get stuck in stable equilibria, some of which are circled in electronic supplementary material, figure A1, at intermediate values of *q*. Note that these equilibria can occur either side of the threshold *rb* = *c*. For S recessive, the equilibria occur when *b* < *c*/*r*, for S dominant when *b* > *c*/*r*.

## 4. Mapping the positions of the stable equilibria

In this section, we explore the relationship between stable gene frequencies and the values of *b* and *c* that produce them. We start with the derivation in [17] for full sibs, from the recurrence equations for the genotypic frequencies, of quadratic equations (their eqn 51) that show how equilibrium values of *p* or *q* vary with the values of *b* and *c*. Writing *x* = *b*/*c* − 2, the quadratic equation for the equilibrium values of *p* for S dominant, A recessive is

4.1 $$c{\left(1+x\right)}^{2}p^{2}-c(1+x)p+x=0,$$

and for A dominant and S recessive

4.2 $$c{\left(1+x\right)}^{2}p^{2}-c(1+2x)(1+x)p-x=0.$$

Uyenoyama & Feldman [17] did not distinguish stable from unstable equilibria, but these can be distinguished because the first equilibrium encountered by a favoured allele entering a population is bound to be stable. Thus if there are two internal equilibria, the one with lower *p* is stable for A alleles and the one with lower *q* is stable for S alleles.

**Figure 2.**
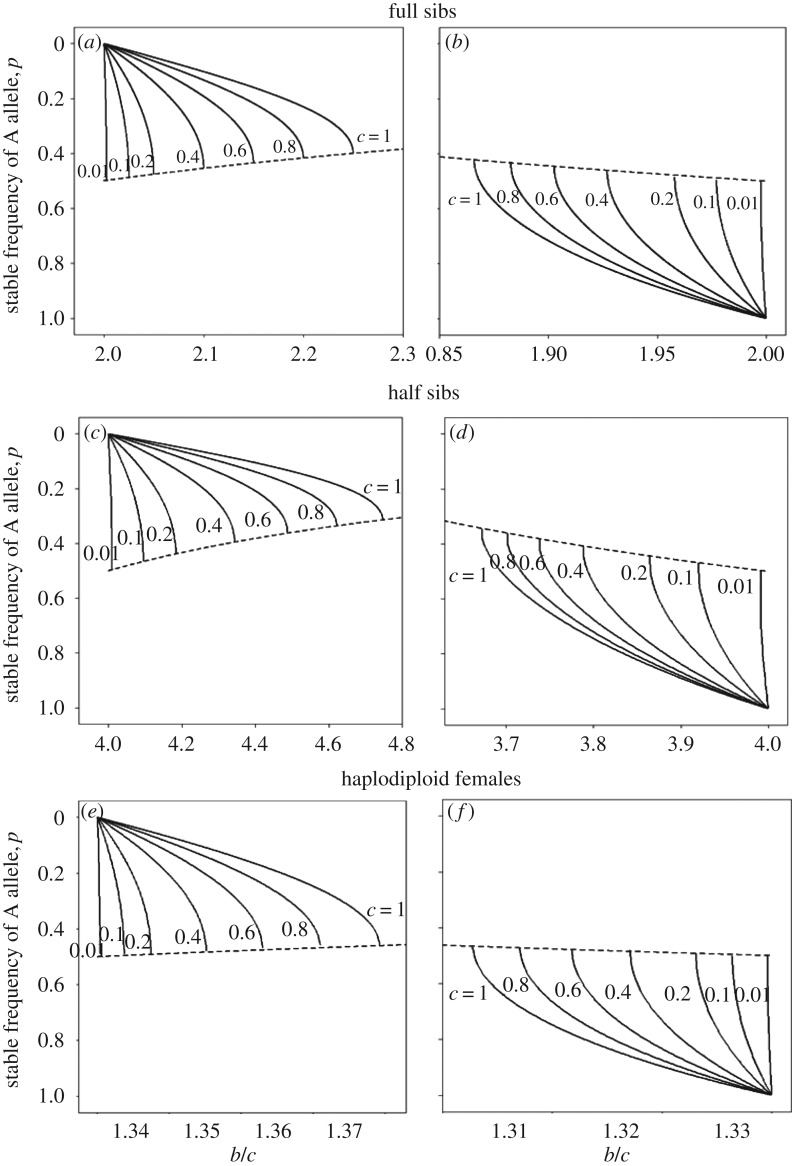
Plots of stable equilibrium values of *p* in relation to values of *b*/*c*, for selected values of *c* (labelled). Dashed line represents the threshold for the occurrence of internal stable equilibria. (*a*,*b*) Full sibs; (*c*,*d*) half sibs; (*e*,*f*) haplodiploid females helping females. Left-hand column: A recessive; right-hand column: A dominant.

Knowing which of the two equilibria of *p* is stable, we can use equations (4.1) and (4.2) to see how the position of the stable equilibrium varies with the values of *b* and *c*. The stable equilibrium values of *p* were found for A recessive using equation (4.1) and are plotted against *b*/*c* in figure 2*a*. Favoured A alleles entering populations homozygous for S spread with *p* increasing to these stable equilibria; figure 2*a* shows how the positions of the stable equilibria depend on the values of *b* and *c*. From figure 2*a*, we see that for a given value of *c*, the stable equilibrium values of *p* increase monotonically from 0 as *b* increases up to greatest divergence from 1 at a point a little below *p* = 0.5. Increasing *b* beyond this point no equilibrium is possible. So the points of greatest divergence represent thresholds for the occurrence of equilibria. If *b* exceeds the threshold then the A allele spreads to fixation, but if the value of *b* is less than the threshold but above 2*c*, the A allele spreads to the stable equilibrium.

The value of *p* is known at the threshold value of *b*/*c*, as indicated by the dashed line in figure 2*a*. The threshold occurs when the roots of the quadratic equation (4.1) become complex at *x* = ¼*c*. The threshold value of *p* has a maximum at *p* = ½ when *b*/*c* = 2, and decreases with *b*/*c* to a minimum at *p* = 0.40 when *c* reaches its maximum plausible value of 1 and *x* = ¼. It is interesting to relate the threshold for the occurrence of equilibria to the curves plotting Δ*q* against *q* in electronic supplementary material, figure A1. For a given value of *c*, the threshold corresponds to a curve with an intermediate maximum which just touches the *x*-axis, i.e. Δ*q* = 0 in electronic supplementary material, figure A1.

So far we have analysed the situation for A recessive in full sibs. Figure 2*b* shows the analogous plot for A dominant. Here, favoured S alleles entering populations homozygous for A spread with *q* increasing (*p* decreasing) up to the stable equilibria; figure 2*b* shows how the positions of the stable equilibria depend on *b*/*c*. The threshold for equilibria decreases as *b*/*c* decreases, from *p* = ½ to *p* = 0.42 when *c* = 1 and *b*/*c* = 1.87, as shown in figure 2*b*.

The stable values of *q* are plotted in similar fashion for half sibs in figure 2*c,d*, and for haplodiploids in figure 2*e,f*. In both cases equations giving the equilibria, the equivalents of equations (4.1) and (4.2), are needed. Uyenoyama & Feldman [17] did not consider the case of half sibs but applying their method to our equation (2.2) gives for half sibs and A dominant the equilibria equation

4.3*a* $${\left(b-2c\right)}^{2}p^{2}-(2b^{2}+8c^{2}-9bc)p-(b-4c)=0.$$

The recurrence equations in the electronic supplementary material give the corresponding equation for A recessive as

4.3*b* $$2{\left(b-2c\right)}^{2}p^{2}-2bcp+2(b-4c)=0.$$

Using these equations, the stable values of *p* are plotted together with the thresholds for existence of stable equilibria for half sibs in figure 2*c,d*. For A recessive (figure 2*c*), the threshold has a maximum at *p* = ½ when *b*/*c* = 4, and decreases with *b*/*c* to *p* = 0.31 when *c* = 1 and *b*/*c* = 4.75. For A dominant (figure 2*d*) the threshold for equilibria decreases from a maximum when *p* = ½ to *p* = 0.34 when *c* = 1 and *b*/*c* = 3.67.

Similar plots for females of haplodiploid species helping sisters can be obtained using quadratic eqn (11) in [17]. For A recessive (figure 2*e*), the equation is

4.4*a* $${\left(b-c\right)}^{2}p^{2}+\frac{b(b-c)}{4p}-\frac{3}{4}b+c=0.$$

The threshold has a maximum at *p* = ½ when *b*/*c* = 4/3 = 1.33, and decreases with *b*/*c* to *p* = 0.46 when *c* = 1 and *b*/*c* = 1.37. For A dominant (figure 2*f*) the equation is

4.4*b* $${\left(b-c\right)}^{2}p^{2}-\left(\frac{7}{4}b-2c\right)(b-c)p-\frac{3}{4}b+c=0.$$

The threshold for equilibria decreases from *p* = ½ to *p* = 0.47 when *c* = 1 and *b*/*c* = 1.31.

## 5. Discussion

Here we have analysed the evolutionary fate of alleles causing their carriers to help full or half sibs or haplodiploid sisters, whose relatednesses are ½, ¼ or ¾, respectively. Our numerical investigation has shown that the conditions for alleles to enter populations are given exactly by Hamilton's pedigree rule, but initially favoured recessive alleles do not spread to fixation if *b* and *c* are close to the threshold *rb* = *c*. Instead they become trapped, producing a stable balance (polymorphism) between selfishness and altruism. The alleles get trapped when alleles lost in disadvantaged genotypes during kin selection are exactly replaced at reproduction by alleles from other genotypes. Investigation of the positions of the stable polymorphisms showed that stable polymorphisms always occur close to the Hamilton threshold *rb* = *c* (figure 2), though it should be borne in mind that if equilibrium allele values are close to 0 or 1 the less frequent allele may be lost through genetic drift. The position of the stable equilibrium moves away 0 or 1 with both increases in *c*, the cost paid by the altruist, and increasing divergence from the Hamilton threshold, and alleles for selfishness can reach frequencies around 50%. The expected frequencies depend critically on the *b*/*c* ratio and the relationship *r* between altruists and recipients.

These findings raise the question of the magnitude of costs, benefits and relatedness in nature. The available evidence, collated and reviewed by Bourke [25], is summarized in table 2.

**Table 2.** Benefits, costs and relatedness in 11 quantitative tests of Hamilton's rule collated and reviewed in [25]. Each row refers to a different species. Bees and wasps are haplodiploid, the others species are diploid. Costs and benefits were measured as number of offspring lost or gained, but are here normalized so that individuals sacrificing all their personal reproduction pay a cost of one; NF is the number by which costs and benefits were divided except NF = 1 where the reported cost was not significantly non-zero. Relatedness, *r*, refers to Hamilton's 1964 [18] pedigree definition but in some cases was averaged over recipients of help. Where a paper contained more than one analysis of Hamilton's rule, only data from the first case are presented here. We were unable to calculate costs and benefits in case 6 of [25] so that case is omitted. Where [25] gives Hamilton's rule in the form *r*_RO_*b* + *r*_O_*c*, we use *r*_RO_/*r*_O_ as the value of *r*. The alternative to the cited behaviour is breeding alone except in the case of the salamander, where the alternative is eating kin. This table is modified from electronic supplementary material, table S1, of [25] which should be consulted for further information.

study animal	behaviour	*b*	*c*	*r*	NF
lace bug	laying in another's nest	13.6	0	0.36	1
bee	guarding shared nest	6.18	1	0.42	0.327
bee	guarding shared nest	2.75	1	1	1.46
bee	helping at nest	0.31	1	1	3.5
wasp	breeding cooperatively	−0.39	1	0.50	3.41
wasp	breeding cooperatively	1.81	1	0.57	12.2
wasp	breeding cooperatively	2.11	1	0.63	1
wasp	breeding cooperatively	28.7	1	0.4	0.184
salamander	eating non-kin	2	0	0.50	1
turkey	cooperative lekking	6.78	1	0.42	0.9
bee-eater	helping at nest	2.81	1	0.66	0.168

In the haplodiploid bees and wasps, the measured values of *r* are high, varying from 0.4 to 1. A value of ¾ is expected for haplodiploid sisters. If these cases related to haplodiploid sisters then polymorphisms would be expected if *b*/*c* is between 1.3 and 1.4 for *c* = 1. Observed values of *b*/*c* are below these values in two of the seven cases, but higher in the other five, suggesting that there all females should help their sisters. The measured values of *r* are high in the turkeys and bee-eaters, being 0.42 and 0.66, respectively. The turkeys are probably full or half brothers, so the observed *b*/*c* ratio of 6.78 exceeds the values where polymorphisms are expected: subdominant male turkeys should help their brothers. The bee-eater helpers include brothers and, not analysed here, some offspring. The observed *b*/*c* ratio of 2.81 exceeds the values where polymorphisms are expected for full brothers, and is below the equivalent region for half-brothers. Given there is imprecision in the quantitative estimates, these analyses suggests that genetic polymorphisms could occur, but none of the cases analysed falls within the regions where polymorphisms are expected from figure 2.

Using the simplest of genetic models, we have identified regions where genetic polymorphisms between selfishness and altruism are expected, but other models also can give rise to genetic polymorphisms. Using methods as here, based on recurrence equations for genotype frequencies, it has been shown that under certain conditions non-additivity of costs and benefits can lead to stable genetic polymorphisms [15], as can discrimination against selfish individuals [16], sexual antagonism acting under balancing selection [26] and heterozygous advantage [14]. Genetic polymorphisms could also arise through selection–mutation balance [27], temporal environmental variation causing selection to act in different directions over time (e.g. [25]), or if altruism was a mixed not a pure strategy [28]. The intermediate degrees of dominance discussed in [12] and [17] could also be studied numerically. A restriction on our method is that the recurrence equations used in our analyses are exact only when the population is large in the sense that there are an infinite number of sibships of each type as defined, for example, in electronic supplementary material, table A1. The evolutionary process is then deterministic. Those limitations have been to some extent overcome in individual-based simulations which showed that Hamilton's 1964 [18] rule accurately predicted the minimum relatedness necessary for altruism to evolve [29]. The individuals evolved in a population of size 1600 and were equipped with haploid genetic systems characterized by mutation, recombination and pleiotropic and epistatic effects. It would be helpful to repeat these experiments with diploidy to replicate the conditions investigated here.

To carry out a test of Hamilton's rule in nature, it is necessary that there exist both selfish and altruistic individuals, whose relative costs and benefits can be compared. But how can such a polymorphism of selfishness and altruism come about? One possibility is that the polymorphism has a genetic basis, so that selfishness and altruism are heritable, as in the cases analysed here. Alternatively, some individuals may be prevented from acting selfishly. This occurs in bee-eaters which choose altruism when the ‘selfish’ option of breeding alone is blocked when their nests are destroyed by close kin [30]; in female insects rendered small by strategic food deprivation whose best option is helping sibs; and in male turkeys unable to achieve dominance within their sibship whose best option is altruistic support of the dominant male [31].

Our results and the experimental work reviewed by Bourke [25] raise the question of whether we should expect to find examples of the coexistence of selfishness and altruism in nature. To answer this, we need to consider how helping evolves over evolutionary time. Suppose genes arise through mutation, which cause their carriers to provide food, protection or support to relatives when needed. At the start of the evolutionary process it may be that this help confers benefits at a cost to helpers such that *rb* − *c* > 0, but *rb* − *c* may not be in the region where polymorphisms are expected to occur. In this case, the genes responsible would be selected and spread through the population to fixation. However, there may still be further benefits available at some additional cost to the helper. Eventually further benefits are only available by pushing the benefits into the region at which stable polymorphisms are expected to occur. Recessive genes that cause their carriers to provide/withhold help are then expected to spread initially but to get trapped, as we have shown using the simplest of genetic models. The expected outcome is a stable balance between selfishness and altruism. This together with non-additivity [15], discrimination against selfish individuals [16] and other factors mentioned above may contribute to the substantial heritability of selfishness/altruism found in man. As further genes for altruism are identified, it will be interesting to see whether they are accompanied by alternate alleles for selfishness.

## Supplementary Material

The ESM give the working needed to derive the recurrence equations linking the genotype frequencies in successive generations, and Fig. A1 which shows how the effects of selection can be visualised.

## Data accessibility

Working needed to derive the recurrence equations (equations (2.1*a*–*c*)) together with an additional figure showing how the effects of selection can be visualized are available in the electronic supplementary material.
