# Supplementary material for: Genetic polymorphisms between altruism and selfishness close to the Hamilton threshold rb = c
Source: R Soc Open Sci. 2017 Feb 8;4(2):160649. doi: 10.1098/rsos.160649 (PMC5367295; doi:10.1098/rsos.160649)
Supplement: The ESM give the working needed to derive the recurrence equations linking the genotype frequencies in successive generations, and Fig. A1 which shows how the effects of selection can be visualised. [file rsos160649supp1.pdf]

Supplementary material

Here we give the working needed to derive the recurrence equations (equations 2.1) linking the genotype frequencies in successive generations for the case of half sibs with A dominant. Later we give the corresponding equations and the tables used to derive them for half sibs with A recessive and for full sibs. The frequency of the AA genotype before selection is denoted U, after selection U'. The frequency of the AS and SS genotype are similarly denoted V and V' and W and W'. Then in Fig. A1 we show how these equations can be used to visualise the effects of selection on alleles with specified effects.

<u>*Half sibs with the A allele dominant*</u>

Table A1 shows the values of quantities needed to calculate the expected change in genotype frequencies between two successive generations for the case of half sibs with A dominant. The seven rows of Table A1 correspond to the seven sets of individuals that share the same genotypes and fitnesses. These sets are indexed by j as shown in the first column. The fitness of an individual depends on its parent's genotype because that affects the genotypes of its sibs and these in turn affect the frequency of altruism in the sibship, shown in column 6. The fitness of individuals is shown in the last column of Table A1 (these fitness are not yet normalised by mean fitness, i.e. their weighted sum ≠ 1, this is done later). The fitness of individuals in the first row (j = 1) is calculated by noting that all the sibs in the sibship have an A allele because their common parent is of AA genotype, so their fitness is 1 + b – c, from Table 1. The calculation is harder if the common parent is AS. For j = 3 for example the frequency of altruism in the sibship is ½(1+p) so the individual's fitness is 1 + ½(1+p) (b–c) – ½(1-p) c = 1 + ½(1+p) b – c. To normalise the fitnesses the values in the final column of Table A1 need to be divided by their weighted sum, where the weights are the g_i values. This weighted sum equals 1 + (b-c)(1 - q^2). The weighted sum of genotype fitnesses equals mean fitness and it is worth noting that the mean fitness of the population is always 1 + (b-c)(proportion of altruists in the population). This is because the fitnesses of individuals are only affected when they interact with an altruistic relative. In interactions between two altruistic individuals both the fitnesses are increased by (b-c). In interactions between altruistic and selfish individuals the altruist pays a cost c and the selfish individual receives a benefit b and therefore the average change in fitness is again (b-c).

Table A1. Values of quantities needed to calculate in equations 2.1 the genotype frequencies after selection for half-sib families. The A allele is here assumed to be dominant to the S allele. U, V and W are the frequencies of the genotypes AA, AS and SS in the common parents before selection. The last column gives the unnormalised fitness of the individual from which relative fitness v_j is obtained as explained in the text.

			Frequencies				
J	Parental genotype	Individual genotype	Parental genotype	Common parent and individual g_j	Altruism in sibship	Selfishness in sibship	Individual fitness
1	AA	AA	U	Up	1	0	1+b-c
2	AA	AS	U	Uq	1	0	1+b-c
3	AS	AA	V	V½p	½(1+p)	½(1-p)	1+½(1+p) b-c
4	AS	AS	V	V½	½(1+p)	½(1-p)	1+½(1+p) b-c
5	AS	SS	V	V½q	½(1+p)	½(1-p)	1+½(1+p) b
6	SS	AS	W	Wp	P	q	1+pb-c
7	SS	SS	W	Wq	P	q	1+pb

Half sibs with the A allele recessive

Table A2. Values of quantities needed to calculate U', V' and W' for half-sib families with the A allele recessive to the S allele. U, V and W are the frequencies of the genotypes AA, AS and SS in the common parents. The last column gives the unnormalised fitness of the individual from which relative fitness v_j is obtained.

			Frequencies				
J	Parental genotype	Individual genotype	Parental genotype	Common parent and individual g_j	Altruism in sibship	Selfishness in sibship	Individual fitness
1	AA	AA	U	Up	P	1-p	1+bp-c
2	AA	AS	U	Uq	P	1-p	1+bp
3	AS	AA	V	V½p	½p	1-½p	1+½bp-c
4	AS	AS	V	V½	½p	1-½p	1+½bp
5	AS	SS	V	V½q	½p	1-½p	1+½bp
6	SS	AS	W	Wp	0	1	1
7	SS	SS	W	Wq	0	1	1

The recurrence equations linking the genotype frequencies in successive generations are:-

$$U' = (p^2 + p^2 b(U+V/4) - p^2 c) / (1 + (b-c) p^2) \quad \text{(A1a)}$$

$$V' = (2pq + pb(Uq+V/4)) / (1 + (b-c) p^2) \quad \text{(A1b)}$$

$$W' = (q^2 + pbqV/4) / (1 + (b-c) p^2) \quad \text{(A1c)}$$

Full sibs with the A allele dominant

Table A3. Values of quantities needed to calculate U', V' and W' for full-sib families with the A allele dominant to the S allele. U, V and W are the frequencies of the genotypes AA, AS and SS in the common parents. The last column gives the unnormalised fitness of the individual from which relative fitness v_j is obtained.

			Frequencies			
j	Parental genotype	Individual genotype	Common parent and individual g_j	Altruism in sibship	Selfishness in sibship	Individual fitness
1	AA x AA	AA	U^2	1	0	1+b-c
2	AA x AS	AA	UV	1	0	1+b-c
3	AA x AS	AS	UV	1	0	1+b-c
4	AA x SS	AS	2UW	1	0	1+b-c
5	AS x AS	AA	$V^2/4$	¾	¼	1+¾b-c
6	AS x AS	AS	$V^2/2$	¾	¼	1+¾b-c
7	AS x AS	SS	$V^2/4$	¾	¼	1+¾b
8	AS x SS	AS	VW	½	½	1+½b-c
9	AS x SS	SS	VW	½	½	1+½b
10	SS x SS	SS	W^2	0	1	1

The recurrence equations linking the genotype frequencies in successive generations are:-

$U' = (p^2(1 + b – c) – bV^2/16) / (1 + (b-c) (1 - q^2))$ (A2a)

$V' = (2pq(1 + b – c) – bV(V + 4W)/8) / (1 + (b-c) (1 - q^2))$ (A2b)

$W' = (q^2 + b(3V^2/16 + VW/2) / (1 + (b-c) (1 - q^2))$ (A2c)

Full sibs with the A allele recessive

Table A4. Values of quantities needed to calculate U', V' and W' for full-sib families with the A allele recessive to the S allele. U, V and W are the frequencies of the genotypes AA, AS and SS in the common parents. The last column gives the unnormalised fitness of the individual from which relative fitness v_j is obtained.

			Frequencies			
J	Parental genotype	Individual genotype	Common parent and individual g_j	Altruism in sibship	Selfishness in sibship	Individual fitness
1	AA x AA	AA	U^2	1	0	1+b-c
2	AA x AS	AA	UV	½	½	1+½b-c
3	AA x AS	AS	UV	½	½	1+½b
4	AA x SS	AS	2UW	0	1	1
5	AS x AS	AA	$V^2/4$	¼	¾	1+¼b-c
6	AS x AS	AS	$V^2/2$	¼	¾	1+¼b
7	AS x AS	SS	$V^2/4$	¼	¾	1+¼b
8	AS x SS	AS	VW	0	1	1
9	AS x SS	SS	VW	0	1	1
10	SS x SS	SS	W^2	0	1	1

The recurrence equations linking the genotype frequencies in successive generations are:-

$U' = (p^2 + b(pU + V^2/16) - cp^2) / (1 + (b-c) p^2)$ (A3a)

$V' = (2pq + bV(U + p)/4) / (1 + (b-c) p^2)$ (A3b)

$W' = (q^2 + bV^2/16) / (1 + (b-c) p^2)$ (A3c)

Use of above equations to visualise the effects of selection on alleles with specified effects

In Fig. A1 we provide diagrams like Fig. 1B that allow visualisation of the effects of selection on alleles whose carriers confer benefits and experience costs that can lead to polymorphisms. The diagrams are for half and full sibs. The top three rows of Fig. A1 show the situation for half sibs when costs are 1, 0.1 or 0.01, in rows 1 – 3 respectively. The bottom three rows show analogous results for full sibs. The situation for S recessive is shown in the panels on the left hand side, and their interpretation is analogous to that provided above for Fig. 1B. The situation for S dominant (A recessive) is shown in the panels on the right hand side, and again inspection of whether Δq and Δp are positive or negative shows whether S and A alleles spread or decline.

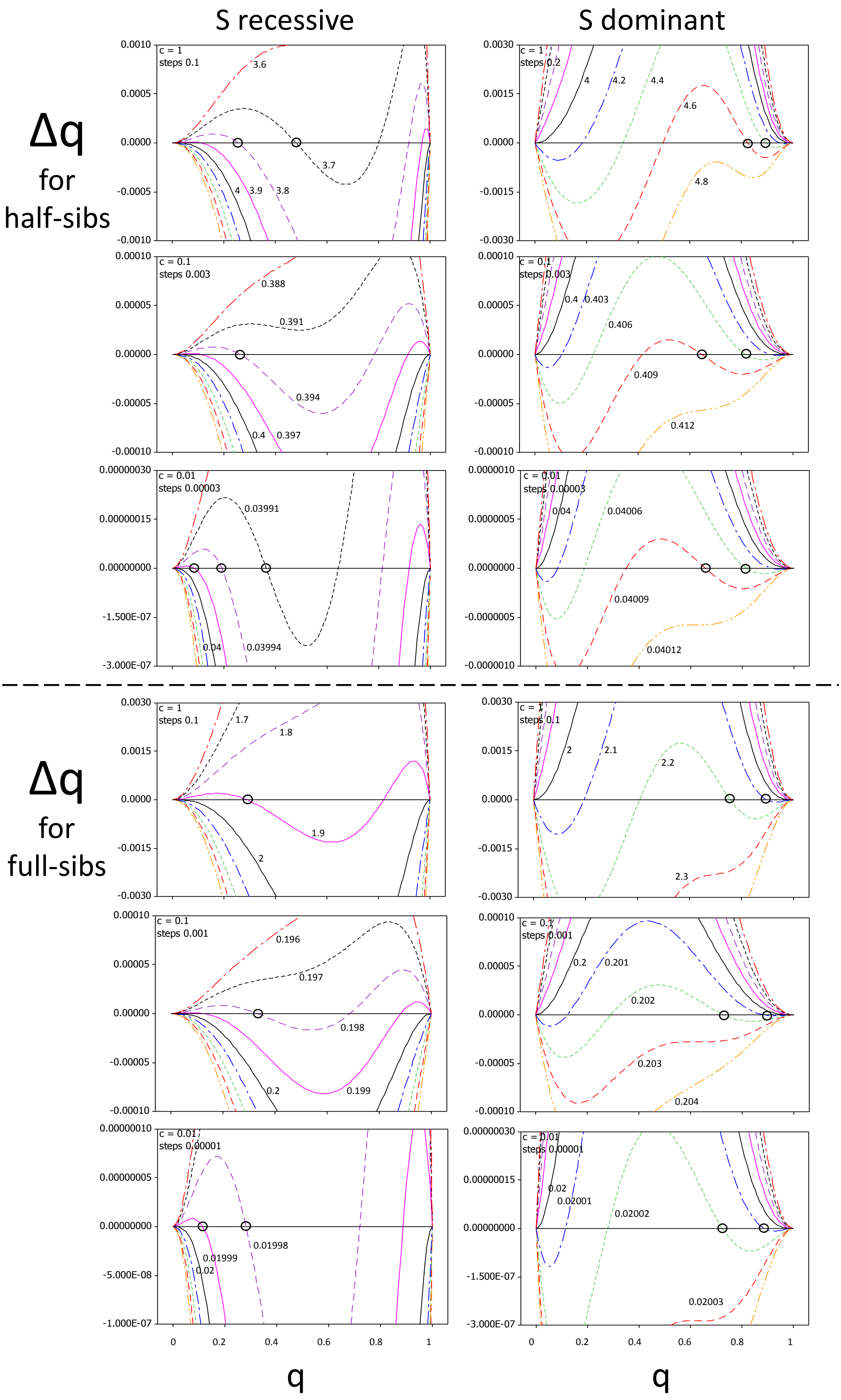

S recessive
S dominant
Δq for half-sibs
Δq for full-sibs
c = 1
steps 0.1
3.6
3.7
3.8
3.9
4
steps 0.2
4.2
4.4
4.6
4.8
c = 0.1
steps 0.003
0.388
0.391
0.394
0.397
0.4
0.403
0.406
0.409
0.412
c = 0.01
steps 0.00003
0.03991
0.03994
0.04
0.04006
0.04009
0.04012
1.7
1.8
1.9
2
2.1
2.2
2.3
steps 0.001
0.196
0.197
0.198
0.199
0.2
0.201
0.202
0.203
0.204
steps 0.00001
0.01998
0.01999
0.02
0.02001
0.02002
0.02003
q

Fig. A1. Plots of Δq vs q for selected values of b and c for half and full sibs. The panels on the left show the situation for S recessive, those on the right for S dominant. The top three rows are for half sibs, the bottom three for full sibs. Each panel refers to a single value of c, shown in upper left of the panel. In each panel the rate of spread of the S allele, Δq, is plotted against the frequency of the S allele, q, for nine values of b. Selected curves are labelled with their b values. The curve corresponding to the threshold for Hamilton's rule is shown as a solid black line, $b = 4c$ for half sibs, $b = 2c$ for full sibs. The other curves increase or decrease the value b in steps of size shown in the upper left of each panel. Equilibria occur where curves intersect the horizontal axis and these may be either stable or unstable. Selected stable equilibria are circled. Note that these equilibria can occur either side of the threshold $r b = c$. In the left-hand panels the equilibria occur when $b < c/r$, in the right-hand panels when $b > c/r$. The spread of the A allele can be analysed using the same graphs by remembering that $p = 1 - q$ and $\Delta p = - \Delta q$.
